# Single‐Cell Analysis Delineates Glutathione Metabolism–Related Gene Signatures in the Glioblastoma Microenvironment and Identifies GSTA4 as a Regulator of Malignant Behaviors

**DOI:** 10.1155/ijog/7575181

**Published:** 2026-04-13

**Authors:** Baozhi Feng, Jie Wang, Wei Shang, Huihao Ma, Yan Fu, Hongtao Lv, Yinghui Xu, Bin Dong

**Affiliations:** ^1^ Department of Neurosurgery, First Affiliated Hospital of Dalian Medical University, Dalian, Liaoning, China, dlmedu.edu.cn; ^2^ Department of Stem Cell and Clinical Research, First Affiliated Hospital of Dalian Medical University, Dalian, Liaoning, China, dlmedu.edu.cn; ^3^ Department of Cardiovascular Imaging, First Affiliated Hospital of Dalian Medical University, Dalian, Liaoning, China, dlmedu.edu.cn; ^4^ The Administration Center, China Medical University, Shenyang, Liaoning, China, cmu.edu.tw

**Keywords:** glioblastoma, GSH metabolism, GSTA4 gene, single-cell RNA sequencing, tumor microenvironment

## Abstract

**Background and Objective:**

The cellular distribution characteristics and functional mechanisms of glutathione (GSH) metabolism within the tumor microenvironment (TME) of glioblastoma multiforme (GBM) remain poorly understood. This study aims to elucidate the cellular landscape and GSH metabolic features of the GBM microenvironment and to clarify the role of GSH S‐transferase alpha 4 (GSTA4) in GBM progression.

**Methods:**

ScRNA‐seq was performed on 104,789 GBM cells to systematically identify cell subpopulations and construct cell interaction networks. The pseudotime analysis was conducted on 50 GSH metabolism–related genes. Differential expression of GSTA4 in GBM cell lines (U87 MG, LN229, and U251) and normal human astrocytes (SVG p12) was validated by quantitative PCR (qPCR) and Western blotting. Subsequently, siRNA and plasmid vectors were employed to establish cellular models with GSTA4 overexpression or knockdown. The functional impact of GSTA4 expression on cell proliferation, invasion, and migration was systematically evaluated. Finally, rescue experiments confirmed that GSTA4 modulates GBM cell function via the Wnt/β‐catenin signaling pathway.

**Results:**

ScRNA‐seq analysis identified 10 major cell types, including tumor cells, proliferating cells, immune cells, and stromal cells. Elevated GSH metabolic activity was predominantly observed in proliferating cells. The pseudotime analysis indicates that GSTA4 exhibits high expression levels in the intermediate state of tumor cells. Functional experiments confirmed significant upregulation of GSTA4 in GBM cells. Silencing GSTA4 expression suppressed cell proliferation, invasion, and migration, whereas its overexpression enhanced these malignant phenotypes. Moreover, this study is the first to demonstrate that GSTA4 promotes tumorigenicity and aggressive phenotypes in GBM cells through the Wnt/β‐catenin signaling pathway.

**Conclusion:**

This study reveals the reprogramming characteristics of GSH metabolism in the GBM microenvironment at the single‐cell level. Furthermore, functional experiments demonstrate that GSTA4 acts as an oncogenic driver in GBM. Collectively, these findings advance the mechanistic understanding of GBM and identify GSTA4 as a promising therapeutic target for precision intervention.

## 1. Introduction

Glioblastoma multiforme (GBM; WHO grade 4 glioma) is the most common primary malignant brain tumor in adults, characterized by exceptionally high invasiveness and recurrence rates [1, 2]. The annual incidence rate of GBM is approximately 3.2 cases per 100,000 individuals, and it significantly increases with age [3]. The current main treatment modalities include surgery, radiotherapy, and chemotherapy, but patient prognosis remains unsatisfactory, with a 5‐year survival rate of less than 10% [4, 5]. Studies have demonstrated that the molecular classification of GBM is closely associated with patient prognosis, and glioma patients harboring IDH mutations and 1p/19q codeletions tend to exhibit a relatively favorable clinical outcome [6–8]. Therefore, it is essential to explore new therapeutic targets and mechanisms in GBM research.

In recent years, studies have demonstrated that the metabolic characteristics in the tumor microenvironment (TME) are closely related to tumorigenesis and cancer progression [9]. Metabolic reprogramming has received extensive attention, as it serves as an important mechanism for cells to adapt to the TME and maintain rapid proliferation [10, 11]. Tumor cells change their metabolic patterns to utilize nutrients, such as glucose, within the body to meet their rapid proliferation needs. This metabolic process not only involves classical aerobic glycolysis (commonly known as the “Warburg effect”) but also includes metabolic reprogramming of amino acids and fatty acids, thereby influencing the malignant biological behavior of tumors [12–14].

Currently, scholars widely recognize the critical role of glutathione (GSH) metabolism in tumor cells. As a key endogenous antioxidant, GSH plays essential roles in maintaining redox homeostasis and regulating signal transduction, and protects cells from damage induced by reactive oxygen species (ROS), lipid peroxides, and cytotoxic electrophilic compounds [15, 16]. However, in the context of cancer biology, GSH exhibits a dual role: While it protects normal cells from oxidative stress, it concurrently promotes the adaptive survival of tumor cells [17]. In malignant tumors, dysregulation of the redox state not only affects tumor cell viability and proliferation but also modulates immune cell infiltration and functional activity within the TME [18, 19]. Tumor cells can modulate intracellular redox homeostasis through alterations in GSH metabolism, thereby influencing their response to oxidative stress. For instance, dysregulation of GSH metabolism affects intracellular ROS levels, which in turn influence cell survival and proliferation [20]. Furthermore, GSH metabolism not only supports tumor cell growth and survival but is also closely linked to resistance against chemotherapeutic agents [21]. Evidence indicates that elevated GSH levels in tumor cells are associated with drug resistance in ovarian, lung, prostate, and colorectal cancers [22].

Although numerous studies have established the pivotal role of GSH in regulating proliferation and apoptosis across a diverse array of tumor cell types, the specific involvement of GSH metabolism–related genes in GBM remains incompletely characterized. This study investigates the expression patterns of GSH metabolism–related genes within the GBM TME, elucidating the features of GSH metabolism across distinct cell types at single‐cell resolution. Furthermore, although elevated expression of GSH S‐transferase alpha 4 (GSTA4) in GBM has been previously reported, its precise regulatory mechanisms in this context remain unclear [23]. Currently, the function of GSTA4 in GBM cells and its association with the Wnt/β‐catenin pathway have not been verified through in vitro experiments. Through comprehensive biological analyses, this study confirms the upregulation of GSTA4 in GBM and demonstrates its significant role in modulating the proliferation, invasion, and migration of GBM cells. Given the great potential of single‐cell sequencing for targeted therapy [24], our findings provide theoretical underpinnings for the development of targeted therapeutic strategies against GBM.

## 2. Methods

1. Single‐Cell Data Analysis: Initially, we obtained 50 classic GSH metabolism genes from the public database (https://www.gsea-msigdb.org/gsea/msigdb). We acquired three single‐cell RNA sequencing (scRNA‐seq) datasets of GBM from the Gene Expression Omnibus (GEO) database: GSE162631, GSE223063, and GSE235676. These three datasets cumulatively provided single‐cell data of 22 GBM samples. We used the CreateSeuratObject function in the Seurat package to read these samples, with the conditions of min.cells = 3 and min.features = 200 set. Subsequently, further quality control was conducted under the following conditions: nCount_RNA ≥ 1000, nFeature_RNA ≥ 200, nFeature_RNA ≤ 8000, and percent.mt ≤ 20. The NormalizeData function was used for data standardization, and the ScaleData function was used for data normalization. The RunHarmony function was used for batch effect removal. The FindClusters analysis was conducted with PC = 20 and resolution = 1.5, and the RunUMAP and RunTSNE functions were used for dimensionality reduction. Each cluster’s cells were defined based on the cell markers. The plot1cell function was used to draw the single‐cell clustering circle plot. Six single‐cell gene set enrichment analysis approaches, including Add, AUCell, UCell, singscore, ssGSEA, and Scoring, were employed to assess the GSH metabolism profile of individual cells [25–27]. Among them, Scoring is a comprehensive score of the previous methods. Intercellular communication analysis was performed using the CellChat package. Given that GSH metabolism exhibited higher activity predominantly in proliferative and tumor cells, these cell populations were selected for in‐depth analysis. Proliferative cells were further refined into three subgroups based on their cluster expression characteristics, namely proliferative tumor cells, proliferative T cells, and proliferative macrophages. Subsequently, we further analyzed the differences in the activity of GSH metabolism characteristics in these three types of cells. Finally, we also conducted pseudotime analysis in proliferative tumor cells and tumor cells, which was based on the monocle algorithm [28].
2. Cell Culture: The SVG p12 cell line was obtained from the Cell Bank of the Chinese Academy of Sciences, and the U251, U87, and LN229 cell lines were purchased from Wuhan Sai Biotech Co., Ltd. The culture medium was composed of 5 mL fetal bovine serum (FSP500, ExCell), 0.5 mL penicillin–streptomycin solution (G4003, Servicebio), and 45 mL DMEM (PM150312, Procell). All media were freshly prepared before use and preheated in a 37°C water bath along with phosphate‐buffered saline (PBS) (G4202, Servicebio) before application. All cell lines were cultured under conditions of 37°C and 5% CO2.
3. Cell Transfection: The siRNA and plasmids were obtained from GenePharma. Transfection should be performed when the tumor cell density in a 9.6 cm^2^ culture plate reaches approximately 60%–80%. The procedure is as follows: System 1 consists of Lipofectamine 2000 (11,668–019, Invitrogen) diluted in Opti‐MEM (A41248‐01, Gibco), followed by incubation at room temperature for approximately 5 min; System 2 contains plasmid DNA or siRNA diluted in Opti‐MEM and is similarly incubated under the same conditions for 5 min. After incubation, Systems 1 and 2 are combined, gently mixed by pipetting, and the resulting complex is incubated at room temperature for 15 min. Immediately thereafter, the transfection mixture is added to the cells. The cells are then cultured in a 37°C incubator for 6 h, after which the medium is replaced with complete medium. Transfection efficiency is evaluated by measuring mRNA expression levels 24 h post‐transfection.
4. Reverse Transcription Quantitative Real‐Time Polymerase Chain Reaction (RT‐qPCR): Following collection of the cell pellet, total RNA was extracted using the SteadyPure Rapid RNA Extraction Kit (AG21023, Accurate Biotechnology [Hunan] Co., Ltd., Changsha, China) according to the manufacturer’s instructions. Subsequently, 20 μL reverse transcription reactions were performed to synthesize cDNA from the purified RNA using the SevenFast Two‐Step RT‐qPCR Kit (SRQ‐01; Seven/Abcells, Beijing, China) according to the manufacturer’s protocol. RT‐qPCR was performed using the SYBR Green method. The primer sequences for GAPDH and GSTA4 are listed in Supporting Table 1. GAPDH was used as the endogenous control gene.
5. Western Blotting: Cells were lysed by incubation with a mixture of phosphatase inhibitors and lysis buffer (GRF103/P0013 B; Epizyme, Shanghai, China) for 30 min on ice. The lysate was vortexed thoroughly to ensure complete homogenization. Following centrifugation at 4°C for 30 min, the supernatant was carefully collected. Protein concentration was determined after incubation at 37°C for 30 min, and a standard curve was generated using a microplate reader to calculate the protein concentration. Equal amounts of protein were separated and subsequently transferred onto a PVDF membrane. The membrane was blocked with 5% skim milk to minimize nonspecific binding. Phosphorylated proteins were blocked using a 5% bovine serum albumin (BSA) solution. After blocking, the membrane was incubated with the primary antibody overnight at 4°C. On the following day, the membrane was incubated with an HRP‐conjugated secondary antibody for approximately 1.5 h at room temperature. Target protein signals were detected using enhanced chemiluminescence reagents. Finally, the resulting images were analyzed to determine the molecular weight and relative expression levels of the target protein.
6. Antibodies: Anti‐GSTA4 (Cat#ab134919, Abcam, the dilution ratio is 1:2000), anti‐β‐tubulin (Cat#80713‐1‐RR, Proteintech, the dilution ratio is 1:10,000), anti‐β‐catenin (Cat#A11932, ABclonal, the dilution ratio is 1:1000), anti‐p‐β‐catenin (Cat#AP1076, ABclonal, the dilution ratio is 1:500), anti‐APC (Cat#A17912, ABclonal, the dilution ratio is 1:2000), anti‐GSK‐3β (Cat#A2081, ABclonal, the dilution ratio is 1:1000), and anti‐cyclin D1 (Cat#A19038, ABclonal, the dilution ratio is 1:1000).
7. Cell Counting Kit‐8: After transfection in a 9.6 cm^2^ culture plate, cells were trypsinized, collected via centrifugation at 1000 rpm for 5 min, and subsequently counted. The cells were then seeded into 96‐well plates at a density of 3000 cells per well and maintained under standard culture conditions (5% CO_2_, 37°C). At 24, 48, and 72 h postseeding, cell viability was assessed using a CCK‐8 assay (ab228554, Abcam). A working solution containing 10% CCK‐8 reagent in culture medium was prepared and incubated at 37°C for 90 min. The absorbance at 450 nm was measured using a microplate reader.
8. Wound Healing Assay: Cells were transfected when confluence reached approximately 80% in a 9.6 cm^2^ culture plate, followed by a wound healing assay. A sterile 1 mL pipette tip was used to create a linear scratch in the center of the monolayer. Subsequently, cells were washed 2–3 times with PBS to remove cellular debris generated during scratching and to minimize interference during image acquisition. To reduce the influence of cell proliferation, the culture medium was replaced with DMEM supplemented with 2% FBS. Cultures were maintained at 37°C in a humidified atmosphere containing 5% CO_2_. Cell migration into the wounded area was monitored and recorded by phase‐contrast microscopy at 0, 24, and 48 h postscratching.
9. Transwell Invasion Assay: Matrigel was diluted with serum‐free DMEM at a 1:8 ratio on ice. A suitable volume of the mixture was added to an 8 μm pore‐size Transwell insert for coating, and the excess liquid was aspirated. The coated insert was hydrated with 50 μL of serum‐free DMEM and incubated at 37°C for 30 min. Prepared cells were resuspended in serum‐free DMEM and carefully seeded into the upper chamber, ensuring even distribution and the absence of air bubbles. The lower chamber was filled with 500 μL of complete culture medium. The assay system was incubated for 48 h. After incubation, the insert was washed with PBS. The cells were fixed with 4% paraformaldehyde for 30 min and then stained with 0.1% crystal violet for 15 min. The insert was washed again with PBS to remove excess dye and air‐dried. Finally, the migrated cells were visualized under a microscope, images were captured, and statistical analysis was performed.

## 3. Results

1. Analysis of Single‐Cell Transcriptional Characteristics and Cell Communication in the GBM Microenvironment: By integrating single‐cell data from 22 samples, we ultimately obtained the transcriptional characteristics of 104,789 cells. These cells were clustered into 39 distinct subpopulations and further annotated into 10 major cell types (Figure 1(a)). The cellular composition included 719 B cells, 4083 endothelial cells, 55,679 macrophages, 7983 monocytes, 3762 oligodendrocytes, 4546 pericytes, 5829 proliferating cells, 6473 T/NK cells, 2307 tumor‐associated neutrophils (TANs), and 13,408 tumor cells. The marker genes of each cell type are shown in Figures 1(b) and 1(c). The results of cell communication analysis are presented in Figure 1(d), indicating that tumor cells have a complex communication network with other cell types in the GBM environment. As shown in Figures 1(e) and 1(f), potential communication pathways between proliferating cells and other cell types, including the SPP1‐CD44 and APP‐CD74 pathways, have been identified.
2. Enrichment Scores of GSH Metabolism–Related Genes in scRNA‐seq: The results of single‐cell enrichment analysis indicated that GSH metabolism was mainly active in proliferative cells, tumor cells, endothelial cells, mononuclear macrophage, etc. (Figure 2(a)). The UMAP plot in Figure 2(b) visually confirmed this issue. Therefore, we subsequently sorted the proliferative cells for analysis and classified them into three major categories: proliferative tumor cells, proliferative T/NK cells, and proliferative macrophages (Figure 3(a)). All proliferative cells highly expressed proliferation‐like markers TOP2A and STMN1 (Figure 3(b)). Meanwhile, proliferative macrophages also highly expressed macrophage markers CSF1R, CD14, and FCER1G (Figure 3(c)). Proliferative T/NK cells highly expressed T/NK cell markers CD3D, CD3E, and NKG7 (Figure 3(d)). Proliferative tumor cells highly expressed tumor cell markers ASCL1, OLIG2, and SOX2 (Figure 3(e)). The metabolism of GSH does not show a high score in all proliferative cells. However, we found that only proliferative tumor cells and proliferative macrophages were the main cell sources with high GSH metabolism scores (Figures 2(c) and 2(d)).
3. Pseudotime Analysis and Dynamic Characteristics of Genes Related to GSH Metabolism: To further elucidate the significant role of GSH metabolism–related genes in the disease process, we conducted a pseudotime analysis. The results of the pseudotime analysis based on proliferative tumor cells are shown in Figure 4. Figures 4(a) and 4(b), respectively, reveal the temporal distribution characteristics and cell states. Figure 4(c) reveals the changes in the GSH metabolism score over time. We found that GSH metabolism did not become stronger or weaker with the progression of time, indicating that it has been playing an important role throughout the entire time course. Figure 4(d) reveals the changes in GSH metabolism–related genes over time.
 In addition to proliferative cells, GSH metabolism in tumor cells is also markedly upregulated. To investigate this further, we performed a pseudotime analysis on tumor cells, with results presented in Figure 5. Consistent with previous observations, the overall level of GSH metabolism in tumor cells remains stable over time, exhibiting a high degree of conservation (Figures 5(a), 5(b), and 5(c)). Nevertheless, individual genes involved in GSH metabolism may exert distinct regulatory roles at different stages of tumor progression (Figure 5(d)). The pseudotime analysis indicates that GSTA4 exhibits high expression levels in the intermediate state of tumor cells.
4. In Vitro Functional Experimental Verification of GSTA4 in GBM: To investigate the role of GSTA4 in GBM, we conducted a series of comprehensive molecular biological experiments. Western blot and RT‐qPCR analyses revealed that GSTA4 expression at both the mRNA and protein levels was significantly higher in three GBM cell lines compared to normal human astrocytes (Figures 6(a) and 6(b)). To assess the functional implications of GSTA4 in GBM, we established cell models with modulated GSTA4 expression in U251, U87, and LN229 cell lines. Specifically, GSTA4 knockdown was achieved in LN229 and U251 cells using small interfering RNA (siRNA), while GSTA4 overexpression was induced in U87 cells via transfection with an overexpression plasmid. RT‐qPCR analysis confirmed successful modulation of GSTA4 expression (Figure 6(c)). Given that proliferation, invasion, and migration are key malignant phenotypes of tumor cells [29–31], we evaluated each of these cellular behaviors. CCK‐8 assays demonstrated that GSTA4 overexpression in U87 cells promoted cell proliferation, whereas GSTA4 knockdown in LN229 and U251 cells suppressed proliferative capacity (Figure 6(d)). Transwell invasion assays indicated that GSTA4 overexpression enhanced invasive potential in U87 cells, while its downregulation reduced invasiveness in LN229 and U251 cells (Figure 6(e)). Furthermore, wound healing assays were conducted [32]. The results showed that GSTA4 overexpression accelerated migration in U87 cells, whereas GSTA4 silencing attenuated migratory ability in LN229 and U251 cells (Figure 6(f)). GSEA indicated that GSTA4 exhibits regulatory potential with respect to the Wnt/β‐catenin signaling pathway (Figure 7(a)), which is critically involved in the initiation and progression of GBM. Based on these findings, we hypothesized that GSTA4 may promote GBM cell proliferation, migration, and invasion through activation of the Wnt/β‐catenin pathway. To test this hypothesis, we assessed key proteins within the Wnt/β‐catenin pathway via Western blotting. Upon knockdown of GSTA4 in LN299 and U251 cells, expression levels of *β*‐catenin were downregulated, whereas those of p‐β‐catenin, GSK3β, and APC were upregulated (Figure 7(b) and 7(c). Additionally, the expression of cyclin D1, a downstream target of Wnt signaling, was also reduced, further supporting the role of GSTA4 in pathway activation. We sought to determine whether GSTA4‐mediated regulation of malignant biological behaviors in glioma is dependent on activation of the Wnt/β‐catenin signaling pathway. We administered the Wnt pathway inhibitor MSAB to the transfected cell lines. Notably, the migration, invasion, and migration abilities promoted by overexpression of GSTA4 were partially restored (Figure 7(d), 7(e), and 7(f).

.

FIGURE 1Single‐cell transcriptomic profiling and cell–cell communication network analysis in the GBM microenvironment. (a) Single‐cell clustering reveals 10 distinct cell subpopulations. (b and c) Expression profiles of signature genes characterizing each subpopulation. (d) A complex intercellular communication network is observed among the identified subpopulations. (e and f) Proliferative cells interact with neighboring cells via signaling pathways including SPP1‐CD44 and APP‐CD74.

**Figure:**
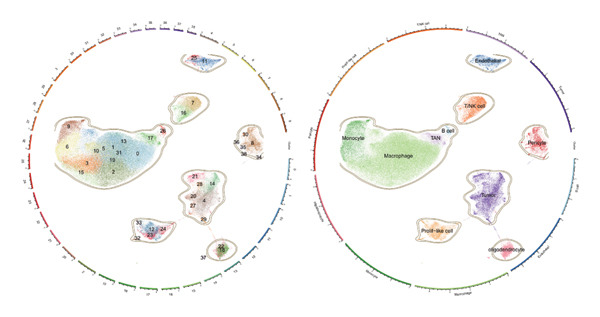
(a)

**Figure:**
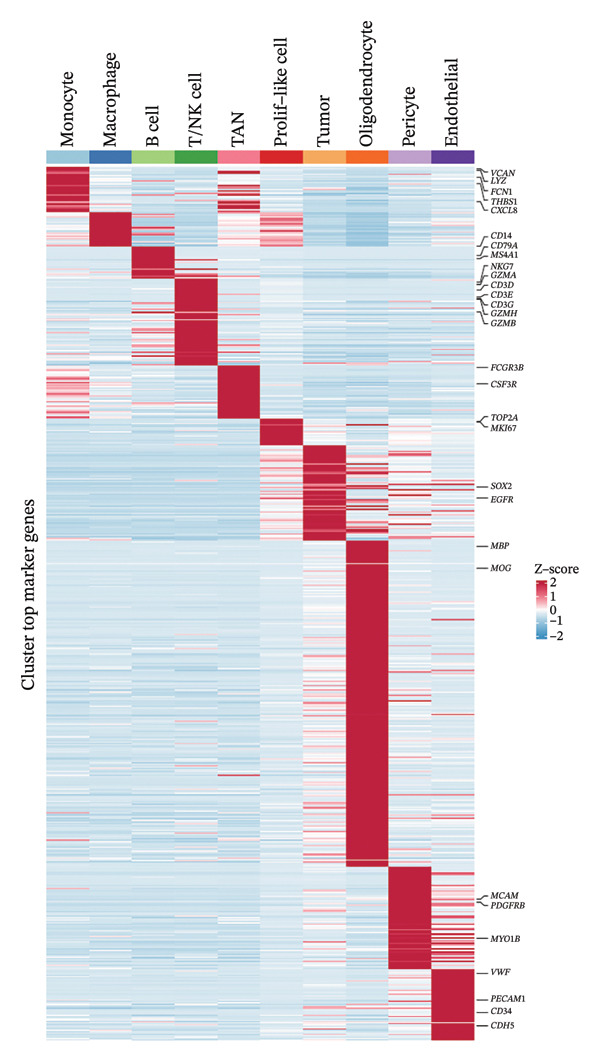
(b)

**Figure:**
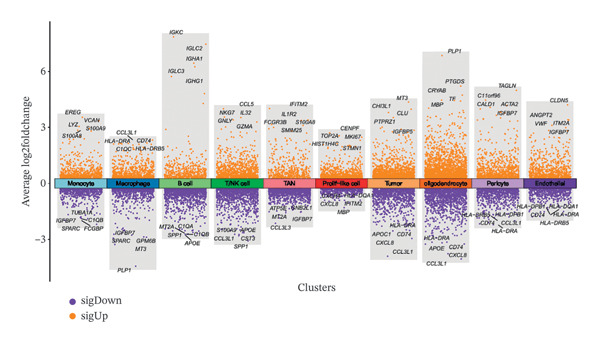
(c)

**Figure:**
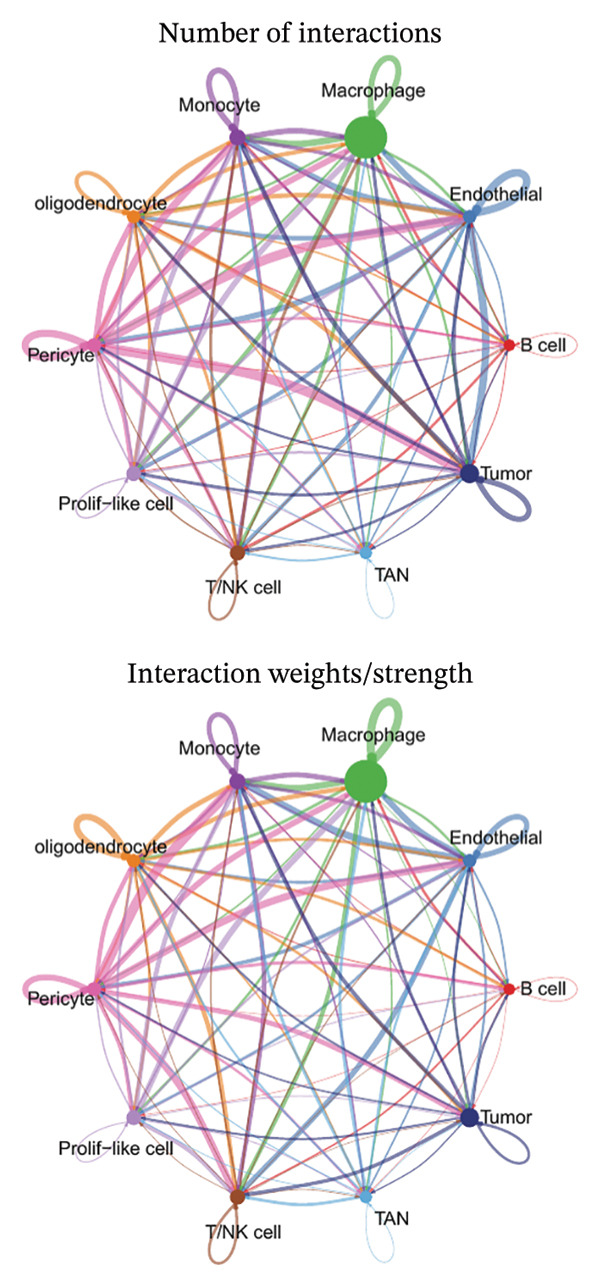
(d)

**Figure:**
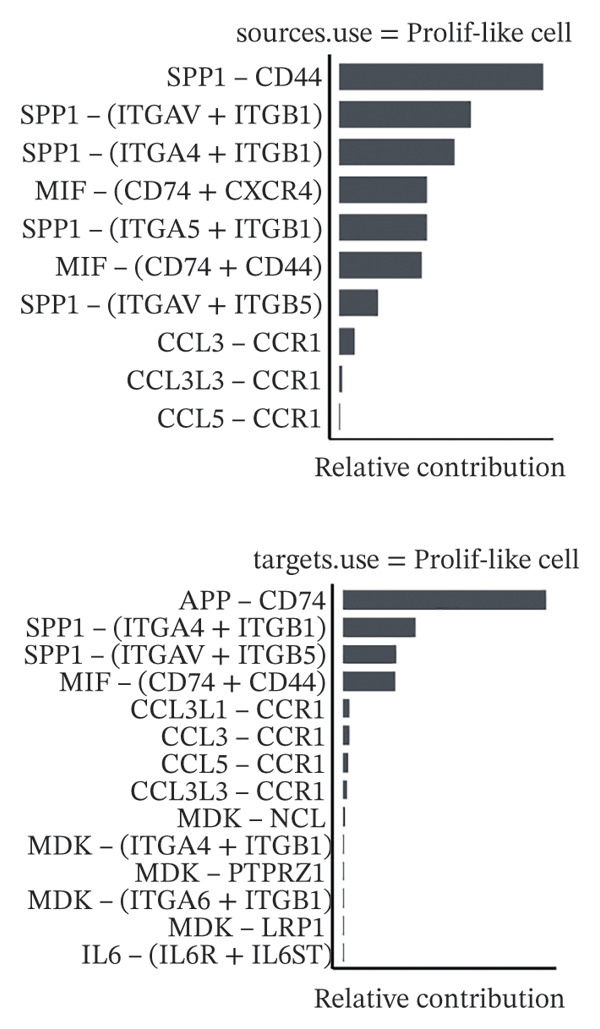
(e)

**Figure:**
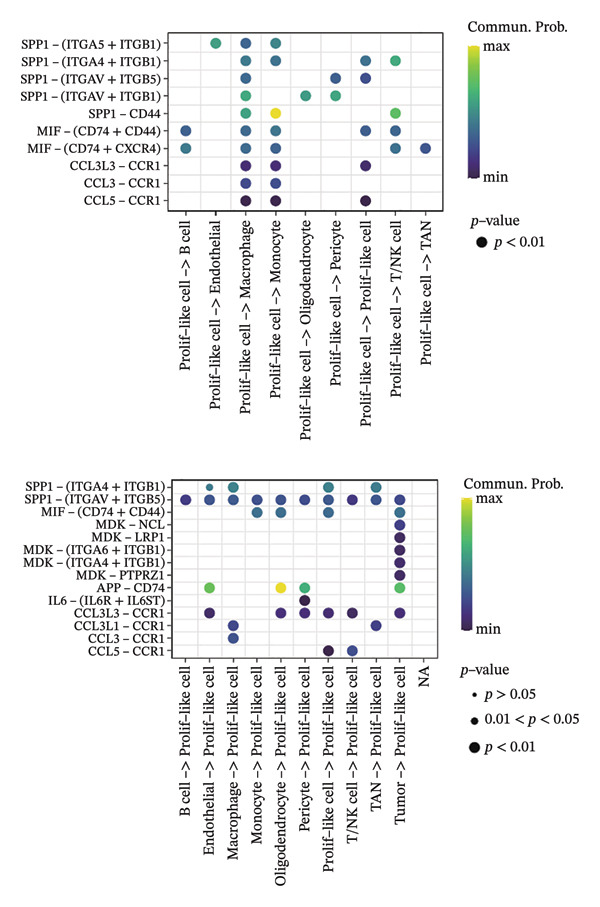
(f)

FIGURE 2Enrichment scores of glutathione‐related genes in single‐cell subpopulations. (a) Scores of six single‐cell gene sets, with the proliferative cell subpopulation having the highest score. (b) UMAP plot of scores of different subpopulation cells. (c) Enrichment scores after sorting of the proliferative cell subpopulation. (d) UMAP plot after sorting of the proliferative cell subpopulation.

**Figure:**
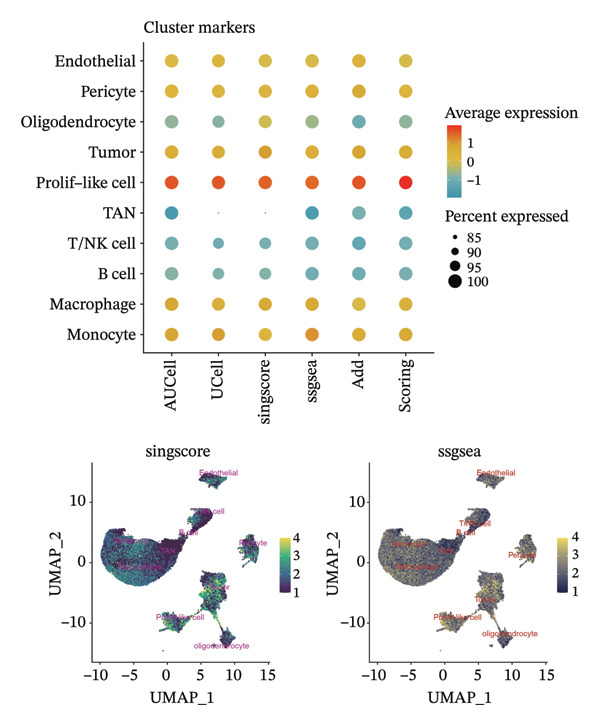
(a)

**Figure:**
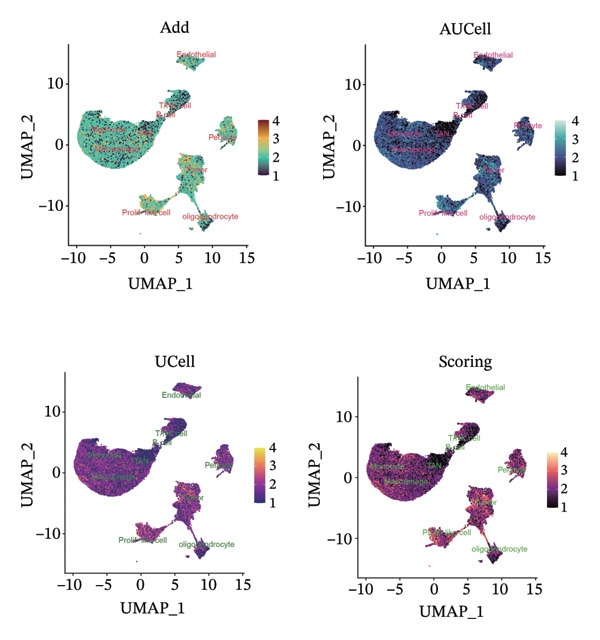
(b)

**Figure:**
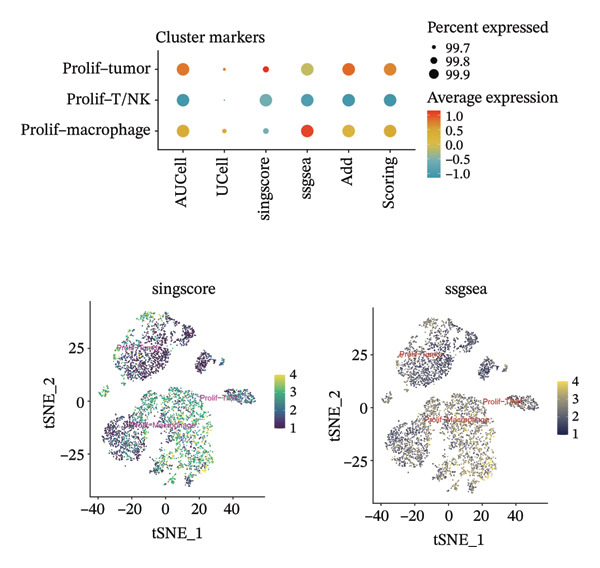
(c)

**Figure:**
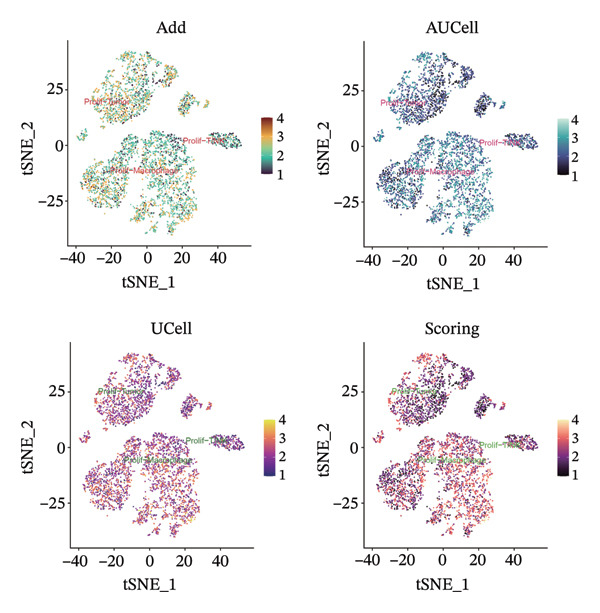
(d)

FIGURE 3Sorting and characterization of proliferative cell subpopulations. (a) Dimensionality reduction map of single cells generated using the t‐SNE algorithm. (b) Proliferative cells exhibit high expression levels of proliferation‐associated markers STMN1 and TOP2A. (c) Proliferative macrophages show elevated expression of canonical macrophage markers, including CSF1R, CD14, and FCER1G. (d) Proliferative T/NK cells display robust expression of T/NK lineage‐specific markers CD3D, CD3E, and NKG7. (e) Proliferative tumor cells demonstrate prominent expression of tumor‐associated markers ASCL1, OLIG2, and SOX2.

**Figure:**
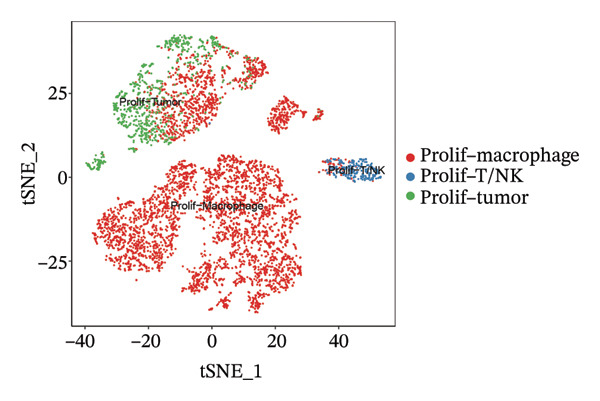
(a)

**Figure:**
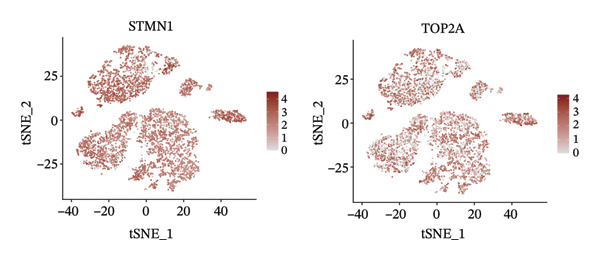
(b)

**Figure:**
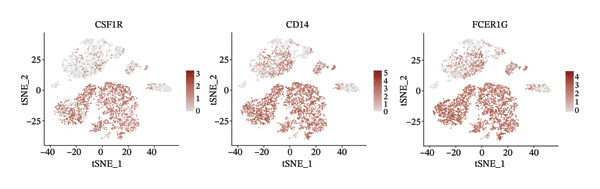
(c)

**Figure:**
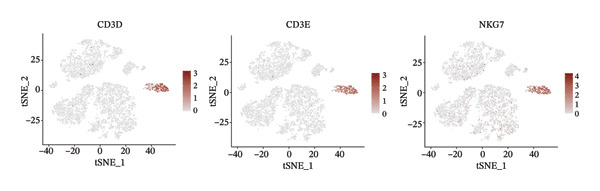
(d)

**Figure:**
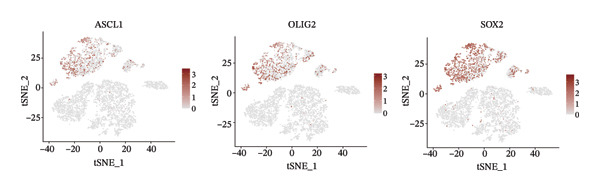
(e)

FIGURE 4Pseudotime analysis of glutathione metabolism–related genes in proliferative tumor cells. (a) Distribution of pseudotemporal ordering. (b) Cell state distribution of proliferative tumor cells. (c) Dynamic changes in the enrichment scores of six single‐cell gene signatures across pseudotemporal progression. (d) Expression dynamics of GSH metabolism–related genes throughout pseudotime.

**Figure:**
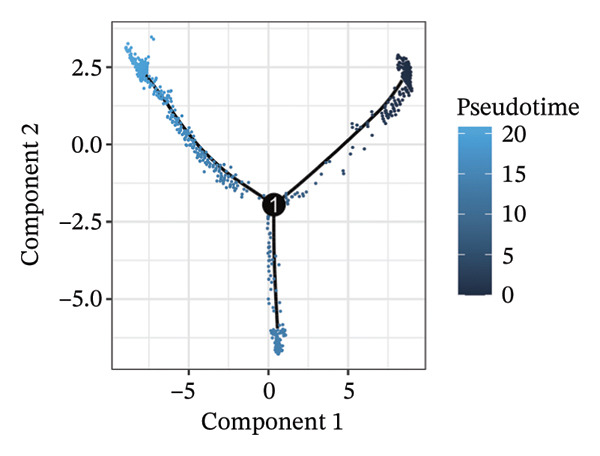
(a)

**Figure:**
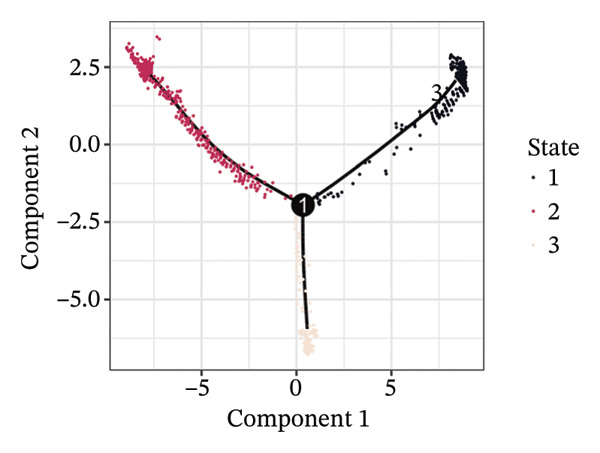
(b)

**Figure:**
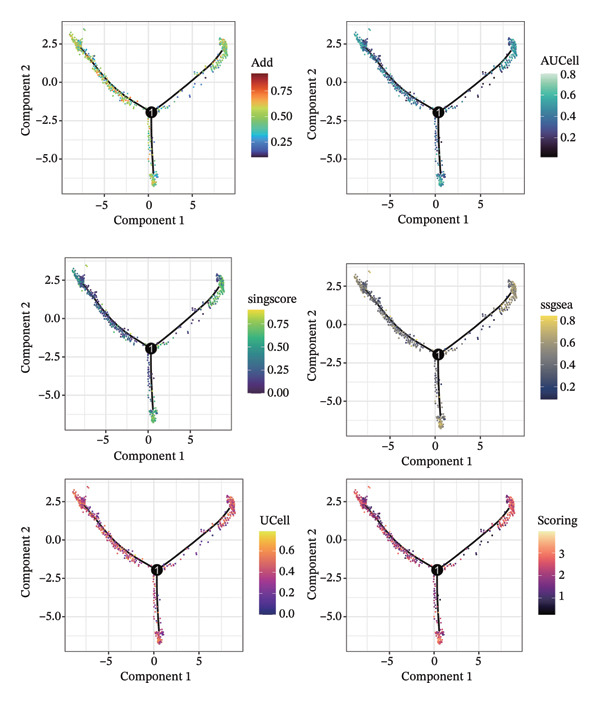
(c)

**Figure:**
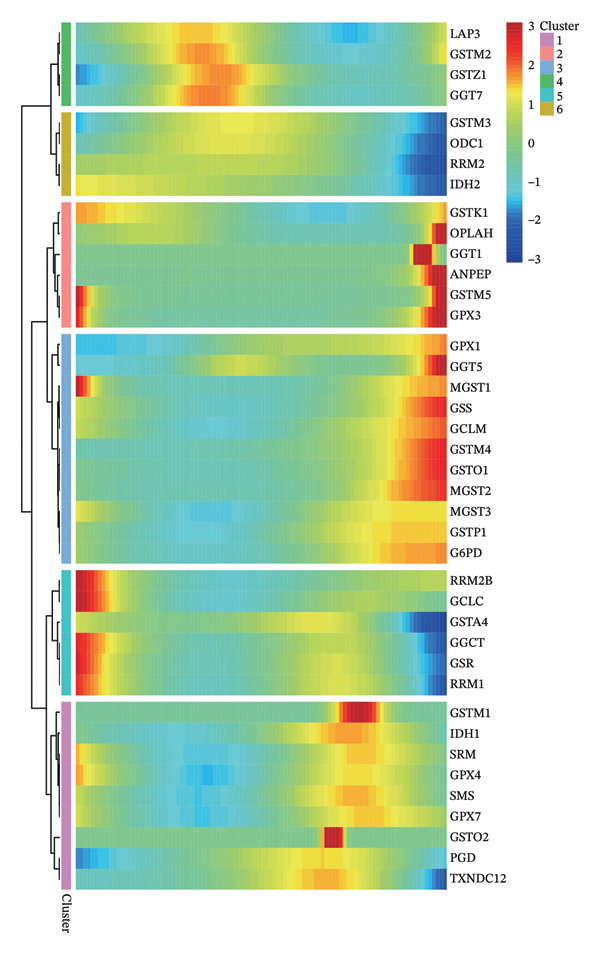
(d)

FIGURE 5Pseudotime analysis of glutathione metabolism–related genes in tumor cells. (a) Pseudotime time distribution. (b) Distribution of tumor cell states. (c) Changes in glutathione metabolism score over time. (d) Changes in glutathione metabolism–related genes over time.

**Figure:**
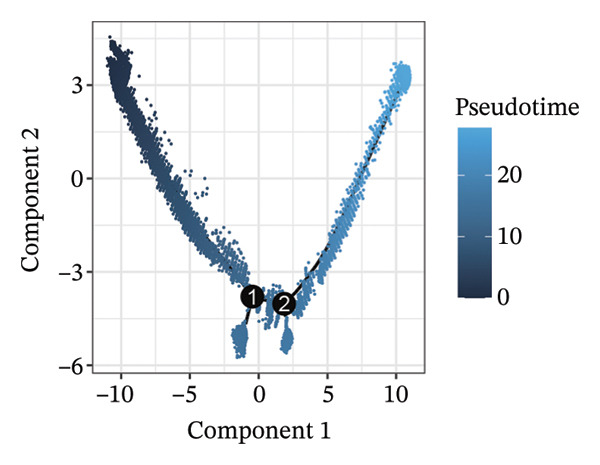
(a)

**Figure:**
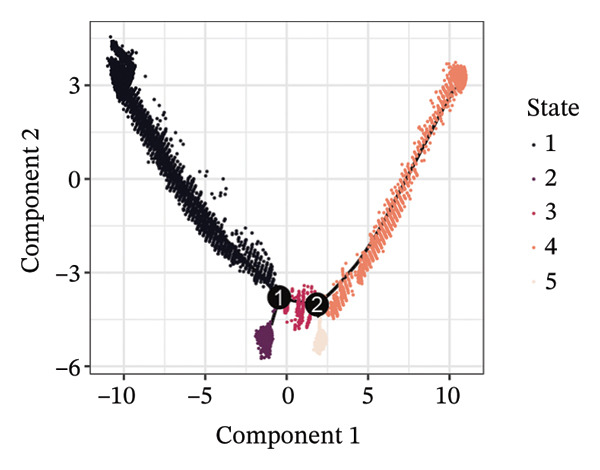
(b)

**Figure:**
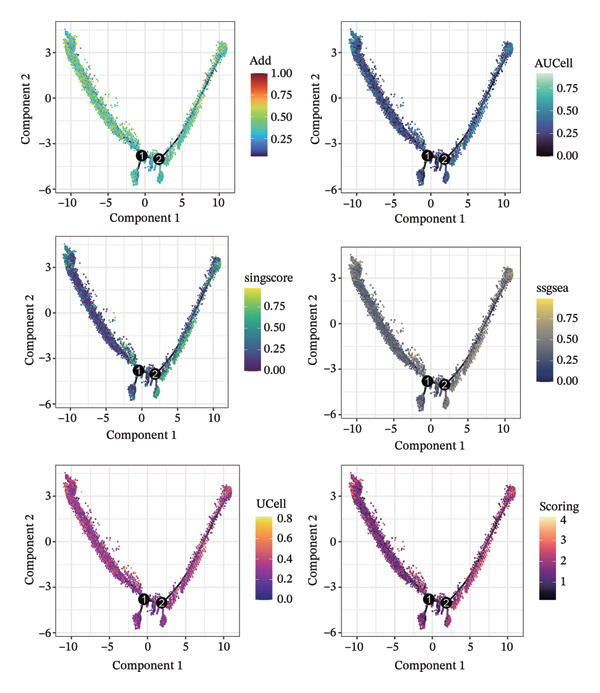
(c)

**Figure:**
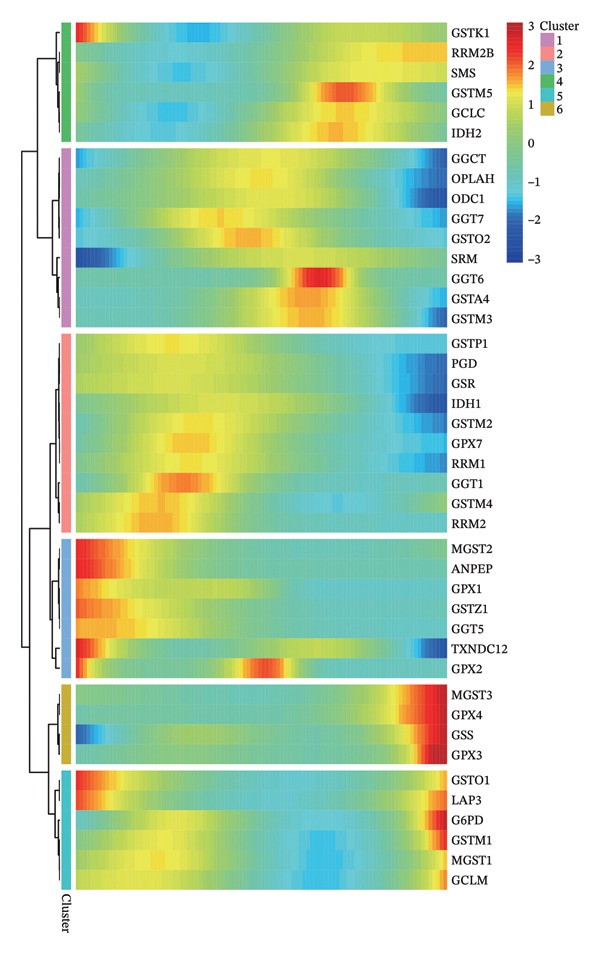
(d)

FIGURE 6Verification of the cellular function of GSTA4 in GBM. (a) GSTA4 is highly expressed at the mRNA level in GBM cells, with relatively higher expression observed in U251 and LN229 cell lines. (b) Elevated GSTA4 protein expression was confirmed in GBM cells. (c) Cell models with modulated GSTA4 expression (overexpression or knockdown) were established, and qPCR analysis validated the corresponding changes in GSTA4 mRNA levels. (d) CCK‐8 assays demonstrated that GSTA4 overexpression enhanced proliferative capacity in U87 cells, whereas its knockdown suppressed proliferation in LN229 and U251 cells. (e) Transwell invasion assays revealed that GSTA4 overexpression promoted invasive potential, while its downregulation exerted an inhibitory effect. (f) Wound healing assays indicated that GSTA4 overexpression increased migratory ability, whereas its suppression reduced migration (^∗^ *p* < 0.05, ^∗∗^ *p* < 0.01, ^∗∗∗^ *p* < 0.001, and  ^∗∗∗∗^ *p* < 0.0001).

**Figure:**
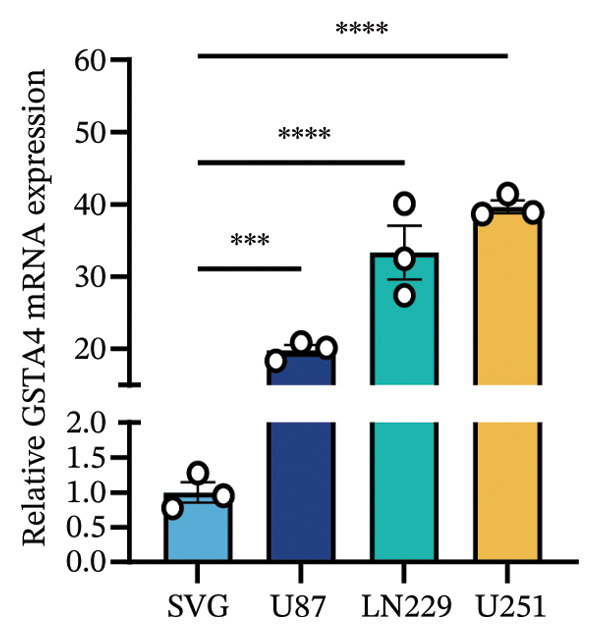
(a)

**Figure:**
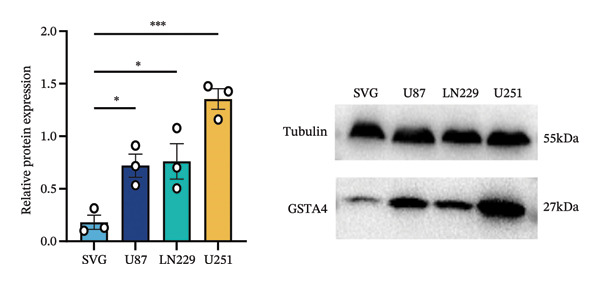
(b)

**Figure:**
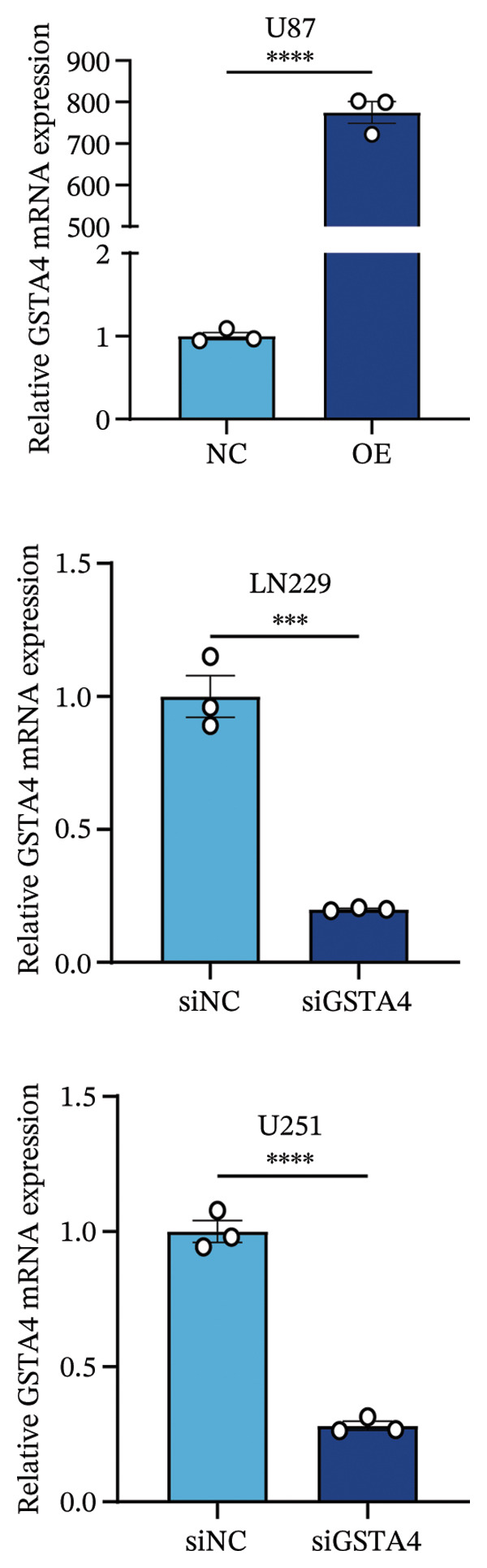
(c)

**Figure:**
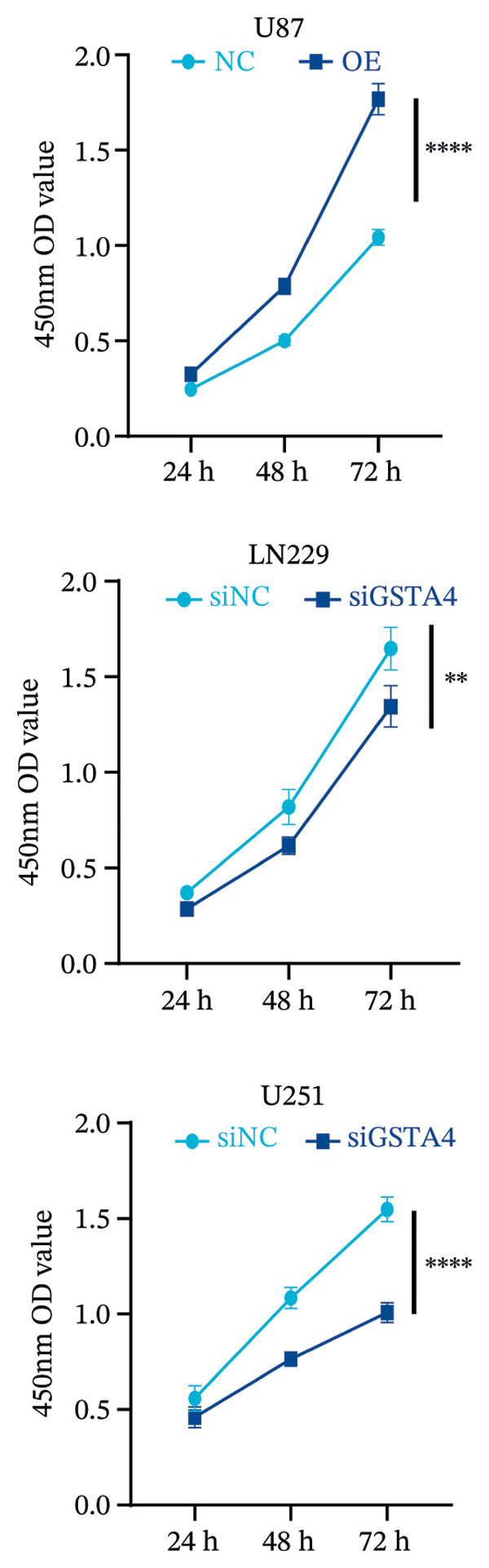
(d)

**Figure:**
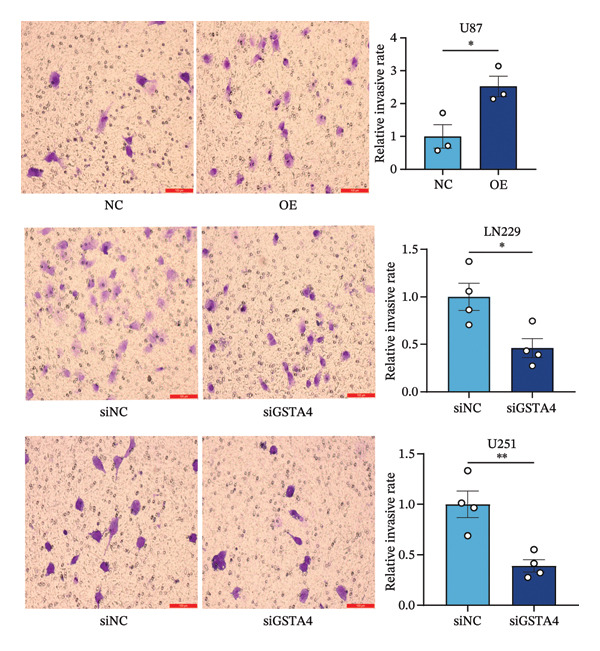
(e)

**Figure:**
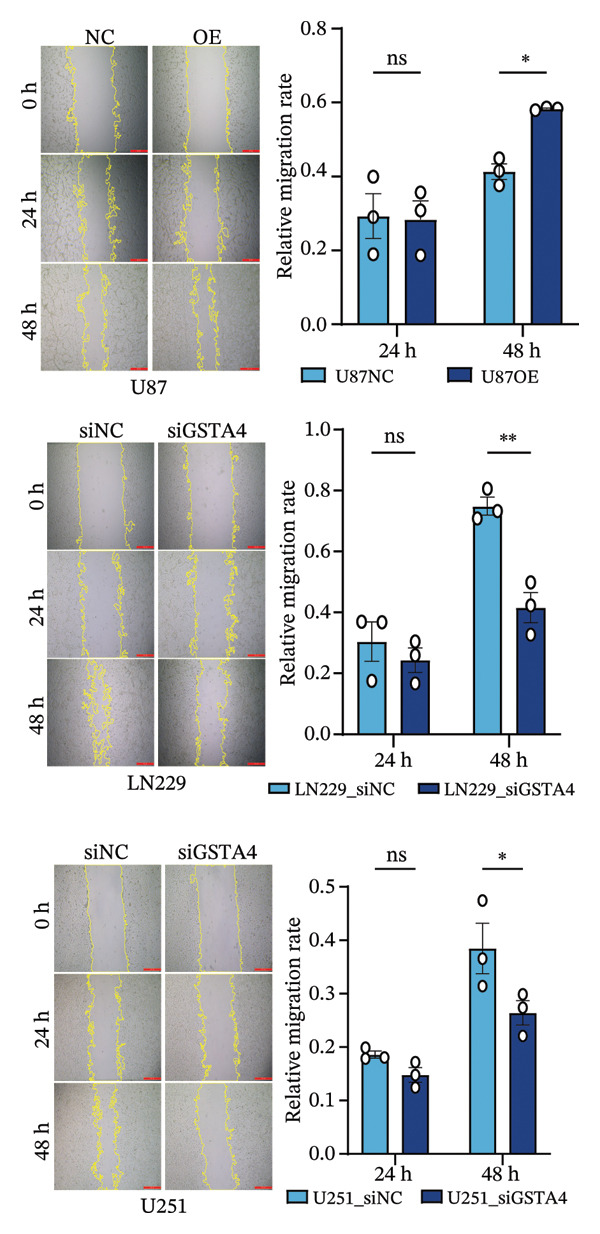
(f)

FIGURE 7The regulatory effect of GSTA4 on GBM cells via the Wnt/β‐catenin signaling pathway. (a) GSEA revealed enrichment of the Wnt‐β‐catenin pathway. (b and c) *β*‐Catenin expression was downregulated, while p‐β‐catenin, GSK3β, and APC expression were upregulated, and the expression of the Wnt downstream target cyclin D1 was downregulated. (d) The CCK8 assay was used to verify the proliferation ability. After overexpression in U87 cells, the proliferation ability was enhanced, and it was weakened after adding the pathway inhibitor. (e) Transwell invasion assay showed that the invasion ability was enhanced after overexpression and weakened after adding the pathway inhibitor. (f) Wound healing assay demonstrated that the migration ability was enhanced after overexpression and weakened after adding the pathway inhibitor (^∗^ *p* < 0.05, ^∗∗^ *p* < 0.01, ^∗∗∗^ *p* < 0.001, and  ^∗∗∗∗^ *p* < 0.0001).

**Figure:**
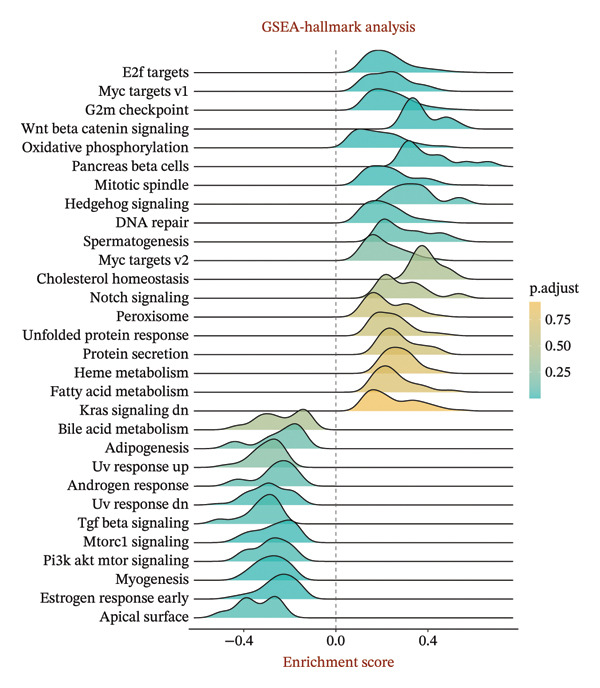
(a)

**Figure:**
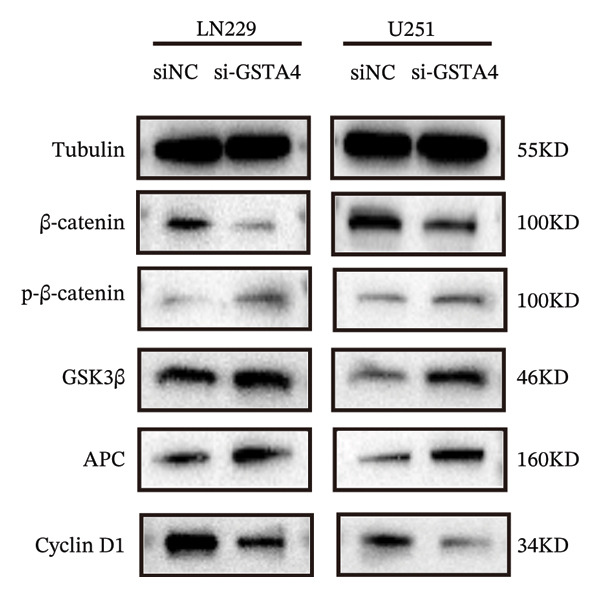
(b)

**Figure:**
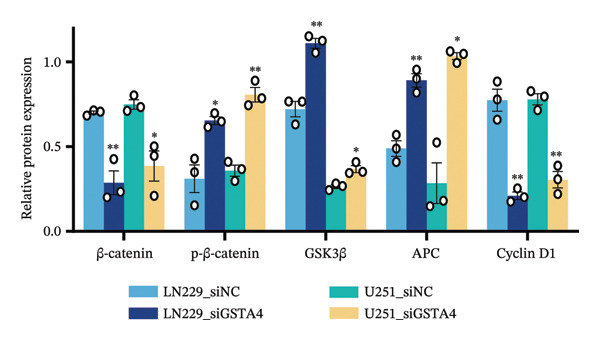
(c)

**Figure:**
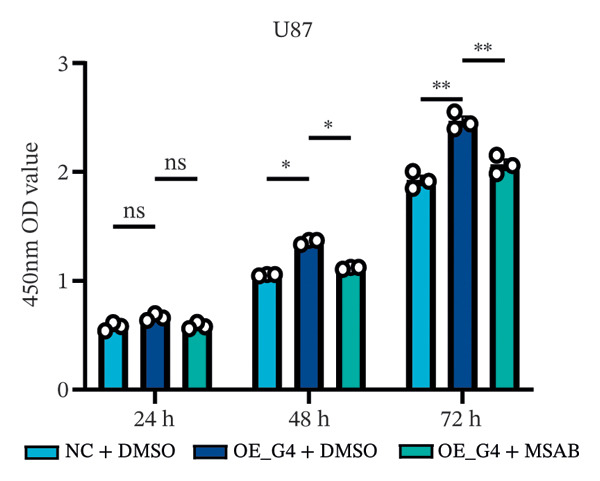
(d)

**Figure:**
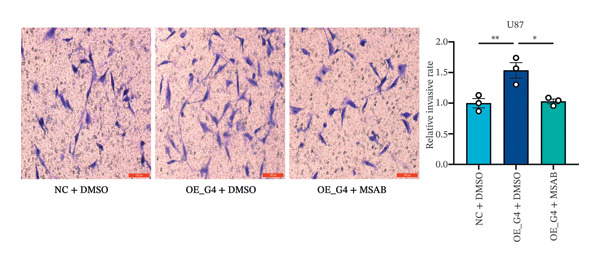
(e)

**Figure:**
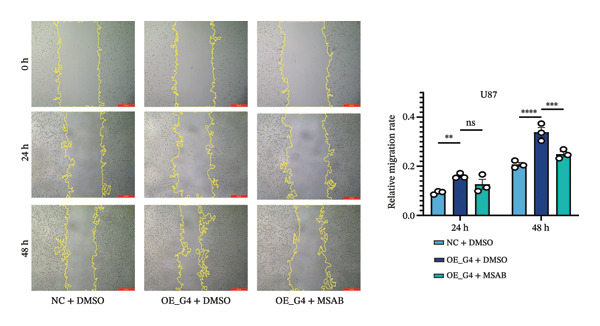
(f)

## 4. Discussion

In this study, we first obtained 50 canonical GSH metabolism–related genes. Using scRNA‐seq data, we systematically analyzed the expression patterns of these genes within the GBM TME. Additionally, we experimentally validated the functional role of the GSH metabolism–associated gene GSTA4 in GBM through a series of molecular biology assays.

Solid tumors consist of diverse cell and extracellular matrix components, exhibiting significant cellular heterogeneity. Consequently, the local milieu surrounding tumor cells forms a complex network [33, 34]. To meet heightened energy demands, tumor cells often exhibit accelerated metabolic activity, and multiple studies have shown that GSH metabolism is involved [35–37]. To protect against ROS‐induced damage, cancer cells elevate intracellular GSH levels, thereby maintaining a prosurvival redox balance conducive to sustained proliferation [38]. Currently, therapeutic approaches targeting tumor cell metabolism to improve the TME have become one of the future research directions in cancer treatment [39]. Therefore, the development of inhibitors targeting GSH will be a future direction for cancer treatment. Notably, suppression of GSH metabolism has been shown to inhibit the progression of various malignancies, including hepatocellular carcinoma, colorectal cancer, and breast cancer [40–42]. However, GSH metabolism within the TME of GBM has not yet been systematically elucidated. In this study, by integrating scRNA‐seq with functional validation experiments, we have, for the first time, uncovered cell‐type‐specific patterns and molecular regulatory mechanisms underlying GSH metabolic reprogramming in GBM at single‐cell resolution. These findings provide novel insights into the metabolic dependencies of GBM and offer a foundation for the development of targeted therapeutic strategies.

Firstly, this study identified 39 cell clusters and 10 major cell subtypes among 104,789 cells, encompassing immune cells (including T/NK cells, macrophages, and neutrophils), stromal cells (such as endothelial cells, pericytes, and oligodendrocytes), tumor cells, and proliferative cells, thereby providing a comprehensive characterization of cellular composition diversity within the glioma microenvironment. Metabolic heterogeneity represents a key feature of the TME, and this study revealed that GSH metabolism in GBM exhibits marked cell subtype specificity. Among the 10 major cell subtypes identified through single‐cell transcriptomic analysis, the GSH metabolism score of proliferative cells is the highest. These findings indicate that proliferative cells serve as the primary executors of GSH metabolism in GBM. From a functional standpoint, proliferative cells undergo rapid cell division, leading to substantial production of ROS. Enhanced GSH metabolism contributes to genomic stability by scavenging excess ROS and sustaining redox homeostasis, thereby supporting sustained cellular proliferation [43].

Secondly, we performed a cell communication analysis and identified a complex network of cellular interactions within the GBM microenvironment, particularly extensive crosstalk between tumor cells and various other cell types. This finding further underscores the critical role of intercellular communication in GBM disease progression. Our analysis revealed that the SPP1‐CD44 and APP‐CD74 signaling pathways may play significant roles in mediating proliferative cell communication. Given the highly metabolic nature of the glioma microenvironment [44], these pathways are likely to contribute to the malignant progression of gliomas. This discovery is consistent with the accumulating evidence indicating that the SPP1‐CD44 axis serves as one of the important molecular hubs driving GBM [45, 46]. Notably, the seminal work by some scholars demonstrated that within the microenvironment of gliomas, SPP1‐CD44 signaling promotes tumor invasiveness and therapy resistance by inducing a stem cell–like phenotype [47]. Similarly, some scholars reported that the APP‐CD74 axis facilitates immune evasion in GBM by suppressing the phagocytic activity of tumor‐associated macrophages (TAMs) [48]. Collectively, these findings support the functional relevance of the SPP1‐CD44 and APP‐CD74 pathways in promoting proliferative and immunomodulatory communication. Furthermore, the elevated GSH metabolism scores observed in proliferative tumor cells and macrophages highlight the heightened metabolic state characteristic of GBM. These pathways play a role in regulating key biological processes, including cell proliferation, invasion, and immune evasion, thereby offering candidate targets for therapeutic strategies targeting the GBM microenvironment. Furthermore, studies have already demonstrated that GSH metabolism in GBM shapes an immunosuppressive TME through multiple mechanisms [49]. We found that the GSH metabolism score was higher in proliferative tumor cells and proliferative macrophages through enrichment scoring, which verified the previous conclusion and indicated that GSH metabolism plays a significant role in regulating the tumor immune microenvironment of GBM. Emerging evidence supports the involvement of both the SPP1–CD44 and APP–CD74 signaling pathways in modulating the immune microenvironment of GBM [48, 50]. Collectively, these findings suggest a potential synergistic role for the SPP1–CD44 and APP–CD74 signaling axes together with the GSH metabolism pathway in driving GBM progression. Further experiments are needed in the future to verify the interaction among these pathways.

GSTA4, a member of the GSH S‐transferase family, has traditionally been recognized for its role in xenobiotic metabolism and defense against oxidative stress [51, 52]. Recent studies have shown that GSTA4 is highly expressed in GBM tissues [23]. However, its specific biological functions and underlying regulatory mechanisms remain poorly understood. This study systematically addresses this knowledge gap. We confirmed the elevated expression of GSTA4 in GBM cells via PCR and Western blotting assays. Functional experiments demonstrate that GSTA4 promotes the proliferation, invasion, and migration of GBM cells, supporting its oncogenic role in GBM pathogenesis. Moreover, we also explored the regulatory mechanism of GSTA4 through cell molecular experiments, especially its dependence on the Wnt/β‐catenin pathway, which is an important innovation of our study beyond previous research. Notably, the study reveals the distinct temporal dynamics of GSTA4 expression through pseudotime analysis. The expression level of GSTA4 peaks during the intermediate stage. This intermediate stage might be a critical window period for GBM cells to complete the malignant phenotypic transformation. Under conditions of chronically elevated ROS, GSTA4 may facilitate cellular adaptation to metabolic stress during phenotypic remodeling by enhancing GSH‐mediated detoxification and redox homeostasis, thereby promoting somatic mutations and oncogenic transformation [53]. Furthermore, our findings imply that GSTA4 may serve as a promising therapeutic target for GBM, and suppression of its expression or enzymatic activity could provide a novel strategy to inhibit tumor invasion and metastasis [54].

This study has certain limitations, as the experiments were primarily conducted using in vitro cell line models. Future studies should incorporate patient‐derived cells and orthotopic xenograft tumor models to validate the functional role of GSTA4 within the in vivo TME. Furthermore, clinical tissue samples should be analyzed to assess the correlation between GSTA4 and *β*‐catenin expression levels, as well as their association with patient prognosis.

## 5. Conclusion

In conclusion, this study utilized single‐cell analysis to provide a novel perspective on the cellular landscape and the regulation of GSH metabolism within the GBM microenvironment. The role of GSH metabolism in the dynamic process of GBM cells was elucidated at single‐cell resolution. Furthermore, the study identifies GSTA4 as a promoter of malignant phenotypes in GBM cells, offering new molecular insights into GBM progression.

## Author Contributions

Baozhi Feng: conceptualization, data curation, formal analysis, methodology, project administration, software, validation, and writing–original draft. Jie Wang: conceptualization, data curation, formal analysis, methodology, project administration, and writing–original draft. Wei Shang: conceptualization, data curation, formal analysis, methodology, project administration, and writing–original draft. Huihao Ma: conceptualization, data curation, and writing–original draft. Yan Fu: data curation, formal analysis, and writing–original draft. Hongtao Lv: conceptualization, project administration, and writing–review and editing. Yinghui Xu: conceptualization, project administration, and writing–review and editing. Bin Dong: conceptualization, funding acquisition, project administration, resources, supervision, and writing–review and editing.

## Funding

The authors have nothing to report.

## Ethics Statement

Publicly available data used in this study can be accessed through the GEO database. As this study did not involve human participants or human tissue, ethical approval and informed consent were not required.

## Consent

Please see ethics statement.

## Conflicts of Interest

The authors declare no conflicts of interest.

## Supporting Information

The primer sequences for GAPDH and GSTA4.

## Supporting information


**Supporting Information** Additional supporting information can be found online in the Supporting Information section.

## Data Availability

The datasets generated and/or analyzed during this study have been deposited in the National Center for Biotechnology Information (NCBI) Sequence Read Archive (SRA) with the following details: (1) GSE162631 corresponding to SRA accession number: PRJNA682432; data access link: https://www.ncbi.nlm.nih.gov/geo/query/acc.cgi?acc=GSE162631; (2) GSE223063 corresponding to SRA accession number: PRJNA924787; data access link: https://www.ncbi.nlm.nih.gov/geo/query/acc.cgi?acc=GSE223063; and (3) GSE235676 corresponding to SRA accession number: PRJNA986889; data access link: https://www.ncbi.nlm.nih.gov/geo/query/acc.cgi?acc=GSE235676 All deposited datasets are in their final form and available for public retrieval and verification.
