# Supplementary material for: Single‐Cell Analysis Delineates Glutathione Metabolism–Related Gene Signatures in the Glioblastoma Microenvironment and Identifies GSTA4 as a Regulator of Malignant Behaviors
Source: Int J Genomics. 2026 Apr 13;2026:7575181. doi: 10.1155/ijog/7575181 (PMC13073078; doi:10.1155/ijog/7575181)
Supplement: Supplementary file 1 — Supporting Information Additional supporting information can be found online in the Supporting Information section. [file IJOG-2026-7575181-s001.docx]

Supplementary Table 1

|  | Forward | Reverse |
| --- | --- | --- |
| GAPDH | ACGGGAAGCTTGTCATCAAT | TGGACTCCACGACGTACTCA |
| GSTA4 | CAGACCCGAAGCATTCTCCAC | AGCAGATCCAGTGTCCCCTC |

The primer sequences for GAPDH and GSTA4.
